# Chromosomal abnormalities: subgroup analysis by maternal age and perinatal features in zhejiang province of China, 2011–2015

**DOI:** 10.1186/s13052-017-0363-y

**Published:** 2017-05-12

**Authors:** Xiao-Hui Zhang, Li-Qian Qiu, Ying-Hui Ye, Jian Xu

**Affiliations:** Women’s Hospital School Of Medicine Zhejiang University, No.1 Xue shi Road, Hangzhou, Zhejiang Province 310006 People’s Republic of China

**Keywords:** Chromosomal abnormalities, Prevalence, Advanced maternal age, China

## Abstract

**Background:**

Recently, the prevalence of chromosomal abnormalities (CA) increased as the increasing proportion of mothers with advanced age. We aimed to explore the prevalence of CA in relation to maternal age and perinatal features.

**Methods:**

A retrospective study was performed based on provincial birth defects surveillance data. The relative risk (RR) and 95% confidence interval (CI) were used to calculate maternal age-specific rates of CA. Socio-demographic characteristics of mothers and perinatal features were listed.

**Results:**

The total prevalence of CA was 6.38 per 10,000 births, which increased per 10,000 births linearly from 4.02 in 2011 to 9.13 in 2015 (*x* ^2^_*line-trend*_ =52.69, *p* < 0.001). During this period, the prevalence for CA per 10,000 births among women over 35 years old increased from 15.34 in 2011 to 33.82 in 2015 (*x* ^2^_*line-trend*_ =115121.6, *p* < 0.001). The RR for overall CA, trisomy 21(T21), trisomy 18(T18) and others in mothers 35 years or older were 6.64 (95% CI 5.55 ~ 7.93), 6.83 (95% CI 5.63 ~ 8.30), 4.06 (95% CI 2.09 ~ 7.90) and 7.54 (95% CI 4.02 ~ 14.11) respectively in comparison to mothers aged 25–29 years old. The stillbirths rate for total CA was 76.45%. T21 and T18 were strongly associated with multiple anomalies, especially congenital heart abnormalities.

**Conclusions:**

The prevalence of CA increased as maternal age increased. Cases with CA were associated with other congenital defects and high mortality risk.

## Background

Chromosomal abnormalities are a significant public health issue due to their adverse impacts on pregnancy outcomes. The most frequent chromosomal abnormalities in newborns have been trisomy-related—such as trisomy 21, 18, and 13—and sex chromosomal abnormalities. Recently, an upward trend in the prevalence of chromosomal abnormalities had been widely reported in relation to the increasing proportion of births to older mothers [1–6]. In Europe, the total prevalence of T21, T18 and T13 all increased over time because of maternal age [1]. In Western Australia, a three-fold increase in the presence of T21 was observed from 1980–2013, corresponding to the steady rise in the number of mothers over 35 years of age [3]. In the Northern Health Region of England, the incidence of T18 and T13 increased together with a rise in the number of mothers over 35 years of age [2]. However, the opposite also has been observed. For example, a population-based study with data from the Welsh Congenital Anomaly Register reported a declining trend in the prevalence of trisomy 13 (T13) in mothers aged 35 and older although the number of mothers over 35 has steadily increased from 1998–2012 [7].

Chromosomal abnormalities are strongly associated with adverse pregnancy outcomes, such as early fetal loss, stillbirths and combined with other congenital birth defects [2, 8, 9]. Approximately half of the fetal tissue of abnormal fetuses and spontaneous abortions exhibited chromosomal abnormalities [10]. Even among live births with chromosomal abnormalities, postnatal survival remained poor [11, 12]. In one cohort study, among infants with T18, 1-year survival was 12.6%; for T13, it was 19.8% [11]. Those infants with T21 who received active interventions still faced an increased risk of mental and motor development delay later in life [13]. Overweight and obesity are also frequently reported in children with T21 [14].

In China, the latest national study showed the percentage of mothers over 35 years of age increased from 2.96% in 1996 to 8.56% in 2007 [15]. Additional, Chinese national incidence of T21 was 3.05 per 10,000 births from 2003 to 2011 [16]. There were notable differences based on rural versus urban populations and maternal age groups. Most studies in China have focused primarily on vulnerable populations or the relation to prenatal diagnosis [6, 10, 17, 18]. Few studies have provide a comprehensive report on the epidemiology of chromosomal abnormalities based on normal population. In November 2013 and 2015, Chinese government ended “only one child” policy, replaced by birth policy- “second child alone” and “universal two children” step by step. The fertility policy encouraged couples to have two babies, which might lead to a potential improvement of mothers with advanced age. Therefore, to explore the associations of chromosomal abnormalities with maternal age and perinatal features should be given a priority.

Zhejiang province is located in the eastern part of China with well-developed economy and high-coverage of prenatal screening. The most recent research in Zhejiang showed that 18.62% children exhibited chromosomal abnormalities among a sample of 4,129 suspected subjects [19]. This study was only conducted in a hospital and based on live births. In Zhejiang province, a hospital-based birth defect surveillance system has been in place for over 30 years. Cases with chromosomal abnormalities were recruited in this network, including both live births and pregnancy terminations due to abnormalities. In this study, we examined the associations between maternal age and perinatal features with chromosomal abnormalities as a means to support medical decisions and health policy.

## Methods

### Study population

This is a retrospective study based on a hospital-based Birth Defect Surveillance System in Zhejiang province. The surveillance system consists of 91 delivery hospitals and covers about one-third of all births in this province. All births in monitored hospitals were assessed within seven days. Cases identified with congenital abnormalities at any gestational age were registered regardless of whether it was a live birth or the pregnancy was terminated due to birth defects. The identification of congenital abnormalities was performed by experts from the medical fields of genetics, obstetrics, pediatrics, pathology and laboratory clinics. Data were collected based on prenatal screening and clinical reports. Quality control was performed annually by local experts each year.

In Zhejiang province, antenatal screening of all pregnant women is routinely offered. In the first-trimester, screening by maternal serum (free beta human chorionic gonadotropin β-hCG), pregnancy-associated plasma protein-A (PAPP-A) and ultrasound marker (the nuchal fold in the fetal neck, NT) are offered. These screenings serve to identify the risk for T21, T18 and other major chromosomal abnormalities. In the second trimester, prenatal screening by human alpha-fetoprotein (hAFP), human chorionic gonadotropin (HCG), estriol (uE3) and ultrasound screening are provided. Amniocentesis and chorionic villous sampling (CVS) are usually conducted at 16–20 weeks of gestation for pregnancies which are considered to be at high-risk for chromosomal abnormalities either because of abnormal results from the prenatal screening, maternal characteristics (including maternal age over 35 years old) or family histories. Non-invasive prenatal testing (NIPT) is available but not widely used as of 2013. Therefore, NIPT is selective to mothers who meet the requirements. All cases with chromosomal abnormalities were diagnosed by karyotype analysis through amniocentesis or newborn serum screening. The data were linked to maternal obstetric and newborn records, and recorded throughout the network. In this study, a total of 818 cases with chromosomal abnormalities were detected during the period from 2011 to 2015. Due to a missing subtype of karyotypes for seven of the cases, only 811 cases were included in this study.

### Statistical analysis

The occurrence of chromosomal abnormalities was calculated by the number of cases for all subtypes of chromosomal abnormalities per 10,000 births (live births and stillbirths). Trends in the prevalence of chromosomal abnormalities were determined by line chi-square tests. The relative risk (RR) and 95% confidence interval (CI) for chromosomal abnormalities were analyzed for the total population and mothers over 35 years of age. Additionally, the presence of abnormal categories between mothers over 35 years of age was compared to mothers ages 25 through 29. All *P*-values below 0.05 were considered significant. SPSS 16.0 software was used to analyze the data.

## Results

### Demographic information

Eight hundred-eleven cases of infants with chromosomal abnormalities were born to a total of 809 mothers, including two sets of twins during this period. The average age of these mothers was 31.83 ± 6.07 years old, ranging from 16 to 47 years old. Among abnormal cases, 328 were female, 429 were male and 54 were of undetermined sex (Table 1).

**Table 1:** Socio-demographic characteristics of mothers (*N* = 809)

Characteristics		Number	Percentage
Minority		5	0.62
Age	<20	3	0.37
	20–24	98	12.08
	25–29	209	25.77
	30–34	214	26.39
	35–47	285	35.14
Living Area	Rural	323	39.83
	Urban	486	59.93
Education	Some/completed primary school	89	10.97
	Junior/senior high school	414	51.05
	College	301	37.11
	Unknown	5	0.62
Gravidity at time of participation in study	1	91	11.22
	2	121	14.92
	3 and more	597	73.61
Parity at time of participation n study	0	229	28.24
	1	404	49.82
	2 and more	176	21.70

### Trends in prevalence of chromosomal abnormalities

The proportion of births born to mothers aged 35 years and older climbed from 8.83% (19, 557/221, 469) in 2011 to 10.08% (26, 608/264,017) in 2015. During 2011–2015, the overall prevalence for chromosomal abnormalities was 6.38 per 10,000 births (811/1, 270, 351). The total prevalence for chromosomal abnormalities per 10,000 births increased in an obvious and linear fashion from 4.02 in 2011 to 9.13 in 2015 (*x* ^2^_*line-trend*_ =52.69, *p* < 0.001). At the same time, the prevalence for chromosomal abnormalities per 10,000 births among women over 35 years old also increased from 15.34 in 2011 to 33.82 in 2015 (*x* ^2^_*line-trend*_ =115121.6, *p* < 0.001). The RR for chromosomal abnormalities proved significantly higher with advanced maternal age in comparison to the general population each year (Table 2).

**Table 2:** Trends in the incidence of chromosomal abnormalities among all mothers and mothers aged 35 years and older over time

Time	General population			Mothers over 35 years population			RR (95% CI)
	No. of births	No. of abnormal cases	Per 10,000 births	No. of births	No. of abnormal cases	Per 10,000 births	
2011	221,469	89	4.02	19,557	30	15.34	3.82(2.53 ~ 5.78)
2012	253,540	136	5.36	22,397	37	16.52	3.08(2.14 ~ 4.44)
2013	266,036	164	6.16	29,309	63	27.47	4.47(3.34 ~ 5.97)
2014	265,289	181	6.82	23,635	67	28.35	4.16(3.15 ~ 5.51)
2015	264,017	241	9.13	26,608	90	33.82	3.72(2.92 ~ 4.73)
Total	1,270,351	811	6.38	115,127	287	24.93	3.91(3.42 ~ 4.48)

### Maternal age-specific rates of chromosomal abnormalities

In general, the prevalence of abnormalities per 10,000 births was 5.42 for T21, 0.42 for T18, and 0.06 for T13, 0.38 for sex chromosomal abnormalities, and 0.11 for structural chromosomal abnormalities. Births to women aged 35 years or older exhibited the highest rate of chromosomal abnormalities according to maternal age. The RR for overall chromosomal abnormalities, T21, T18 and others (including T13, sex-related chromosomal abnormalities and structural abnormalities) in mothers 35 years or older were 6.64 (95% CI 5.55 ~ 7.93), 6.83 (95% CI 5.63 ~ 8.30), 4.06 (95% CI 2.09 ~ 7.90) and 7.54 (95% CI 4.02 ~ 14.11) respectively in comparison to the reference group (aged 25 ~ 29 years old) (Table 3).

**Table 3:** Chromosomal abnormalities according to maternal ages

Maternal age (years)	No. of births	No. of abnormal cases (per 10,000 births)	T21	T18	T13	Sex-related	Structural abnormality
<20	37,539	3(0.80)	3(0.80)	0	0	0	0
21 ~ 24	296,130	98(3.31)	87(2.94)	5(0.17)	0	5(0.17)	1(0.03)
25 ~ 29	553,822	209(3.78)	174(3.14)	19(0.34)	1(0.02)	12(0.22)	3(0.11)
30 ~ 34	268,036	214(7.98)	179(6.68)	13(0.49)	3(0.11)	15(0.56)	4(0.15)
35 ~ 47	114,824	287(24.99)	246(21.42)	16(1.39)	3(0.26)	16(1.39)	6(0.52)
Total	1,270,351	811(6.38)	689(5.42)	53(0.42)	7(0.06)	48(0.38)	14(0.11)

### Perinatal features by specific chromosomal abnormalities

Distribution of prenatal features by specific abnormalities is shown in Table 4. The fatality rate was more common for T18 and sex chromosomal abnormalities than for others. Stillbirths rate of the whole chromosomal abnormalities was 76.45%. T21 and T18 were strongly associated with multiple anomalies. Additionally, congenital heart abnormalities were more prevalent in cases with T21.

**Table 4:** Perinatal features by specific chromosomal abnormalities

Features	T21 (*n* = 689)	T18 (*n* = 53)	T13 (*n* = 7)	Sex-related (*n* = 48)	Structural abnormality (*n* = 14)	Total (*n* = 811)
Fatality (%)	535(77.65)	51(96.23)	6(85.71)	44(91.67)	11(78.57)	647(79.78)
Stillbirths (≥20 weeks GA %)	514(74.60)	47(88.68)	6(85.71)	42(87.50)	11(78.57)	620(76.45)
Multiple anomalies	64(9.28)	5(9.43)	0	0	0	69(8.50)
Congenital heart anomalies	57(8.27)	1(1.89)	0	0	0	57(7.02)

## Discussion

Over the past few years, we have witnessed an upward trend in the incidence of fetal chromosomal abnormalities in Zhejiang province. The average incidence of chromosomal abnormalities reached 6.38 cases per 10,000 births over five years with a large increase between 2011–2015. This was most likely due to the increasing proportion of mothers over the age of 35 years old, which climbed from 8.83% in 2011 to 10.08% in 2015. This finding was strongly supported by studies around the world. According to EUROCAT (European Surveillance of Congenital Anomalies), the proportion of mothers aged 35 years or older increased from 13% in 1990 to 19% in 2009, and accompanied an increase in trisomy-affected pregnancies [1]. A population-based study from the United Kingdom found that pregnancies for both T13 and T18 per 1000 registered births increased from 0.08 to 0.23 and 0.20 to 0.65 respectively, as the percentage of mothers over 35 years increased from 6 to 15% during 1985–2007 [2]. In Western Australia, the rate of fetal Down syndrome pregnancies increased from 1.1 to 2.9 per 1000 births; births for women aged 35+ years increased from 8% to 20% during 1980–2013 [3]. Aside from maternal age, earlier surveillance as well as wide-spread practice of prenatal screening and diagnosis also contributed to the increased identification of chromosomal abnormalities in these studies. The above surroundings were similar to our province.

In our study, women with advanced age were at higher risk of chromosomal abnormalities. In regard to all births, the RR of chromosomal abnormalities tended to increase by nearly four-fold in older women. In comparison to mothers in the 25–29 years old group, the risk for chromosomal abnormalities by subtype analysis among mothers over 35 years was four to seven times greater. The results have been evidenced worldwide, although there were some differences in the determination of advanced maternal age and sample population. In a study conducted in Korean, for every year of age increase, the odds ratio increased by 1.18 times for T21, by 1.18 times for T18, and by 1.16 times for fetal aneuploidies [5]. According to the EUROCAT study, the Poisson model prevalence rate ratio (PRR) estimated for T21, T18 and T13 ranged from 4.1 to 5.5 in mothers aged 35–39 years old compared to mothers aged 25 to 29 years old [1]. In mothers over 40 years old however, the PRP even had a greater increases, which ranged from 10.2 to 18.9 [1]. In contrast to the above studies, no significant associations between some specific chromosomal defects with increased maternal age were also documented. According to the Welsh Congenital Anomaly Register data, unexpected declining trend in trisomy 13 in older mothers were observed as the proportion of mothers aged over 35 years steadily rose [7]. In the Romanian study, there was no correlation between the cases as T18, TX, TXYY, triploidy and advanced maternal age [20]. In Korea study, no significant correlation between T13, Turner’s syndrome, triple X syndrome and maternal age was reported [5]. It was difficult to draw significant conclusions from small samples included in Korean and Romania. In Welsh Congenital Anomaly Register study, they stressed further need for surveillance.

The general average incidence of chromosomal abnormalities in our study was much lower than the corresponding data in European countries, where chromosomal abnormalities accounted for 43.8 of every 10,000 births [21]. As the most common chromosomal abnormality, the total incidence for T21 in the study was 5.42 per 10,000 births, which was less than the estimated rates in Europe (22.0 per 10,000 births), Croatia (7.01 per 10,000 births), comparable to the population prevalence in the United Kingdom (5.9-6.8 per 10,000 births), yet higher than the Chinese national average (3.05 per 10,000 births) [1, 15, 22, 23]. To fully understand the reasons for this differences, variations in data resources and proportion of mothers with advanced age should be considered. In the Europe study, data were extracted from a population-based surveillance network using information on live births, fetal death from 20 weeks of gestation and termination for fetal anomaly. All abnormal cases were diagnosed prenatally up to one year of life. Additionally, the percentage of mothers with advanced age ranged from 13% to 19%; nearly twice as high as the mothers in our study (10.08% in 2015) [1]. In both Croatia and the United Kingdom, a multiple database improved the number of cases included through access to new birth and perinatal death certificates [22, 23]. However, our study was based on hospital registry and follow-up was conducted seven days after birth. We only compared data in birth defect surveillance system and did not explore relationship with other databases. In China, social and geographical inequalities could decrease the national average of chromosomal abnormalities.

T18 syndrome is commonly known as Edwards syndrome. It is caused by the presence of an extra chromosome; 18. The incidence of T18 ranged from approximately 0.50 to 0.70 per 1000 births and about 0.1 per live 1000 births [1, 2, 21, 24]. T13 is usually regarded as the third most common autosomal trisomy followed by T21 and T18. European countries reported 0.1 to 0.2 per 1000 births and 0.1 per 1000 live births for T13 [1, 2]. T18 and T13 represent serious lethal malformations. Yet, some spontaneous abortions with T18 or T13 might not be captured by our surveillance system due to the lack of karyotype analysis. The incidence of sex chromosomal abnormalities was 1% in pregnancies when prenatal diagnosis was performed, with prenatal detection rate below 50% [4, 25]. The fact that newborns without clinics presentation might not have undergone karyotype confirmation even if they possessed sex chromosomal abnormalities. This could result in an underestimation of sex chromosomal abnormalities (SCAs) in our study. The main sex chromosomal abnormality was Turner syndrome (11cases), 47XXY (15 cases), 47XYY (5 cases), and 47XXX (11 cases). The number of structural malformation was minor in this study, and primarily focused on chromosome fragment deletion.

In our study, the overall stillbirths rate was 76.45%. A slightly higher rate of cases with T18, T13 and SCAs suffered fetal death in comparison to T21. According to EUROCAT, termination for chromosomal abnormalities hovered around 70% for T18 and T13, and was 47% for T21. The influence of legal concerns and religious beliefs might partly explain the different birth decisions with regard to the termination of pregnancies that presented abnormalities. We found that 8.50% of the abnormal cases were concurrent with multiple congenital abnormalities. Of these, 7.02% of the chromosomal abnormalities were associated with cardiovascular malformation. In a European study, 80% and 57% of babies with T18 and T13 respectively had a cardiac anomaly [8]. The low amount of T18 and T13 in our study could explain these differences.

## Conclusions

It is the first full-scale study about the clinical epidemiology of chromosomal abnormalities in relation to maternal age, perinatal features in Zhejiang province of China. Since the fertility policy in China has changed, and reproductive habits in various countries around the world have also undergone radical modifications, it is necessary to take into consideration the age-related chromosomal anomalies. The main point of this article is not that the prevalence of births with CA have increased, due to maternal age increase, which is a well established knowledge, but the fact that this should be taken into consideration by operators involved in medical decisions and health-related policy making.

Two limitations in our study should be noted. First, in a hospital-based birth defect surveillance program, cases born outside monitored hospitals or not identified within seven days of birth were not registered. Second, not all mothers who experienced spontaneous abortion necessarily test for chromosomal abnormalities unless they intended to become pregnant. This could lead to an underestimate of the prevalence of total chromosomal abnormalities and early fetal loss. Therefore, the linking of multiple databases, the identification of miscarriages and post-natal cases should be pursued further in future studies.

## Abbreviations

CA: Chromosomal abnormalities
CI: Confidence interval
CVS: Amniocentesis and chorionic villous sampling
EUROCAT: European surveillance of congenital anomalies
hAFP: Human alpha-fetoprotein
HCG: Human chorionic gonadotropin
NIPT: Non-invasive prenatal testing
NT: the nuchal fold in the fetal neck
PAPP-A: Plasma protein-A
PRR: Poisson model prevalence rate ratio
RR: Relative risk
SCAs: Sex chromosomal abnormalities
T13: Trisomy 13
T18: Trisomy 18
T21: Trisomy 21
uE3: Estriol
β-hCG: Beta human chorionic gonadotropin.
